# Lignocellulosic Biomass Transformations via Greener Oxidative Pretreatment Processes: Access to Energy and Value-Added Chemicals

**DOI:** 10.3389/fchem.2018.00141

**Published:** 2018-04-27

**Authors:** Walter Den, Virender K. Sharma, Mengshan Lee, Govind Nadadur, Rajender S. Varma

**Affiliations:** ^1^Department of Environmental Science and Engineering, Tunghai University, Taichung, Taiwan; ^2^Department of Environmental and Occupational Health, School of Public Health, Texas A&M University, College Station, TX, United States; ^3^Department of Safety, Health and Environmental Engineering, National Kaohsiung First University of Science and Technology, Kaohsiung, Taiwan; ^4^Regional Centre of Advanced Technologies and Materials, Faculty of Science, Palacky University, Olomouc, Czechia

**Keywords:** lignocellulosic biomass, depolymerization, biorefinery, greener oxidation, mild pretreatment, life cycle analysis

## Abstract

Anthropogenic climate change, principally induced by the large volume of carbon dioxide emission from the global economy driven by fossil fuels, has been observed and scientifically proven as a major threat to civilization. Meanwhile, fossil fuel depletion has been identified as a future challenge. Lignocellulosic biomass in the form of organic residues appears to be the most promising option as renewable feedstock for the generation of energy and platform chemicals. As of today, relatively little bioenergy comes from lignocellulosic biomass as compared to feedstock such as starch and sugarcane, primarily due to high cost of production involving pretreatment steps required to fragment biomass components via disruption of the natural recalcitrant structure of these rigid polymers; low efficiency of enzymatic hydrolysis of refractory feedstock presents a major challenge. The valorization of lignin and cellulose into energy products or chemical products is contingent on the effectiveness of selective depolymerization of the pretreatment regime which typically involve harsh pyrolytic and solvothermal processes assisted by corrosive acids or alkaline reagents. These unselective methods decompose lignin into many products that may not be energetically or chemically valuable, or even biologically inhibitory. Exploring milder, selective and greener processes, therefore, has become a critical subject of study for the valorization of these materials in the last decade. Efficient alternative activation processes such as microwave- and ultrasound irradiation are being explored as replacements for pyrolysis and hydrothermolysis, while milder options such as advanced oxidative and catalytic processes should be considered as choices to harsher acid and alkaline processes. Herein, we critically abridge the research on chemical oxidative techniques for the pretreatment of lignocellulosics with the explicit aim to rationalize the objectives of the biomass pretreatment step and the problems associated with the conventional processes. The mechanisms of reaction pathways, selectivity and efficiency of end-products obtained using greener processes such as ozonolysis, photocatalysis, oxidative catalysis, electrochemical oxidation, and Fenton or Fenton-like reactions, as applied to depolymerization of lignocellulosic biomass are summarized with deliberation on future prospects of biorefineries with greener pretreatment processes in the context of the life cycle assessment.

## Introduction

The shift from petroleum- to biomass-derived materials appears to be the plausible long-term pathway to ensure sustainable supply of the carbon feedstock for energy and chemical industry (Bairamzadeh et al., 2018). The use of biomass as a sustainable feedstock, however, does not assure a successful transition without adapting processes designed with green chemistry and cost-competitive manufacturing processes. In this regard, the concept of integrated biorefineries encompassing green chemistry principles for production, enhanced energy and material efficiency, reduced waste generation and toxicity, and the increased reusability of the products at the end of their lives, has drawn particular interests and discussion (Clark et al., 2009; De Bhowmick et al., 2017). Greener processes incorporating concepts such as use of heterogeneous catalysis, water-based reactions, environmentally-friendly oxidants substituting for less efficient processes using volatile organic solvents and materials with high environmental burden (Dick et al., 2017; Pelckmas et al., 2017). Similarly, alternative activation methods such as microwave (MW) and ultrasound technologies should replace energy-intensive heating to facilitate efficient chemical reactions.

Identifying routes of production for both energy and value-added chemicals are imperative, and their idealistic pathways have been discussed in numerous reports (Laurichesse and Averous, 2014; Abdelaziz et al., 2016). For example, biomass feedstocks often containing valuable extractable chemicals, of interest to pharmaceutical and agricultural commodity chemicals, can be isolated using greener processes such as supercritical carbon dioxide extraction. The oxidative polymerization of the extract-free lignocellulosic materials can also produce polyfunctional monomeric compounds that can be used as an alternative or replacement for fossil fuel-derived building blocks, including aromatics, amino acids, biofunctional molecules (e.g., lactic acid, succinic acid), and fatty acids. Selective functionalization of natural lignin polymer has been studied to improve its compatibility in composite and copolymer materials (Crestini et al., 2010).

Increased production of biomass for energy, besides being a promising renewable energy source, has several potential benefits in offsetting substantial use of fossil fuels, heightening energy security in regions without abundant fossil fuel reserves, increasing supplies of liquid transportation fuels, and decreasing net emissions of carbon into the atmosphere per unit of energy delivered (Field et al., 2008). However, the over-exploitation of biomass, especially consumed by animals, as alternative energy sources also could threaten food security and generate environmental problems, such as introducing invasive species by the plantation of foreign energy crops. In this context, energy conversion from lignocellulosic biomass as feedstock is particularly attractive because they do not compete with food crops and are abundant in native vegetation. Lignocellulosic feedstock can be derived from discarded agricultural residues and even municipal sludge comprising high organic contents.

The most common forms of bioenergy chemicals using existing technologies are ethanol (biofuel) and methane (biogas). Sugarcane as feedstock can be easily converted to bioenergy through fermentation and distillation because sugar in the form of monosaccharides and disaccharides are both highly digestible. Starch as feedstock (e.g., corn kernels, potatoes) requires enzymatic hydrolysis to convert polysaccharides (glucose polymer in which glucopyranose units are bonded by α-linkages) to monosaccharides (glucose). Of the two components of starch (amylose and amylopectin), amylopectin presents the greater challenge to enzymatic hydrolysis due to the presence of α-1,6-glycosidic branch points which make up about 4–6% of the glucose. Most hydrolytic enzymes are specific for α-1,4-glucosidic branches, yet the α-1,6-glucosidic links must also be broken for complete hydrolysis of amylopectin to glucose.

Cellulose, conversely, is a linear polysaccharide polymer of glucose disaccharide with β-1,4-glucosidic linkages; a cellulose molecule normally consists of a few hundreds to thousands of glucoses. The unbranched cellulose chains are very densely packed via inter-chain hydrogen bonds. Beneficiary of dense packing, cellulose form strong supports for plants in the presence of microfibrils with high tensile strength. Hydrolysis of cellulose is henceforth a critical step for biofuel production to decompose complex organic polymers (proteins, lipids, carbohydrates) into simpler molecules. These molecules can then undergo acidogenesis (converting long-chain fatty acids into volatile fatty acids and sugars), and acetogenesis (converting volatile fatty acids into acetic acid, carbon dioxide and/or hydrogen). The products of acetogenesis provide substrate for methanogenesis, which converts acetic acid into methane and carbon dioxide. Other simple soluble molecules such as amino acids and fatty acids can also be converted into methane by a sequence of fermentative bacteria. For ethanol fermentation, saccharin materials such as glucose, fructose and sucrose are metabolized, normally by yeasts, to produce ethanol and carbon dioxide.

Ironically, as of today, relatively little bioenergy originates from lignocellulosic biomass as compared to feedstock such as starch and sugarcane, primarily due to high cost of production encompassing biomass pretreatment steps to biomass components (i.e., lignin, cellulose, and hemicellulose) and to disrupt the natural recalcitrant structure of these rigid polymers; complex structure of celluloses renders the molecules resistant to biological degradation. The bottleneck step for either biogas or biofuel pathway remains the same: the low efficiency of enzymatic hydrolysis of the feedstock due to the recalcitrant nature of lignin and cellulose. In fact, pretreatment of lignocellulosic raw materials has been considered the second most expensive unit in the biomass-to-energy cost structure (Mosier et al., 2005). In particular, lignin is notoriously resistant to oxidative, hydrolytic, and biological degradation, and is often regarded as a waste fraction of biomass, despite the presence of rich chemical functionalities (Elizabeth et al., 2016). The full valorization of the lignin into energy products or chemical products is dictated by the effectiveness of selective depolymerization of the pretreatment processes. Existing dissolution methods of lignin involve harsh pyrolytic and solvothermal processes abetted with corrosive acids or alkaline reagents. These methods unselectively decompose lignin into many products that may not be energetically or chemically valuable, or even biologically active. Identifying milder and greener processes that can selectively depolymerize lignocellulosic materials, therefore, is a subject of immense interest for exploration. The primary objective of this work, therefore, is to review the studies performed in recent years for the depolymerization via some of the advanced oxidative processes. Section Present Processes for Biomass Conversion Process to Fuel, Refinery, and Pharmaceutical Precursors of the article provides the necessary background pertaining to the functionalities of pretreating lignocellulosic biomass, as well as the modern physical, chemical, and biological pretreatment processes that have been developed or even practiced industrially. Section Greener Oxidative Processes for Biomass Conversion-Selectivity and Mechanism narrows the discussion into oxidative biomass pretreatment processes that are considered both greener and milder, including ozonolysis, photocatalysis, oxidative catalysis, electrochemical oxidation, Fenton and Fenton-like reactions. The last section, intended to provide an outlook of future biorefineries, summarizes the results from life-cycle assessment studies and discusses the interlinking techno-socio-economic impacts of biorefineries.

## Present processes for biomass conversion process to fuel, refinery, and pharmaceutical precursors

**Figure 1:**
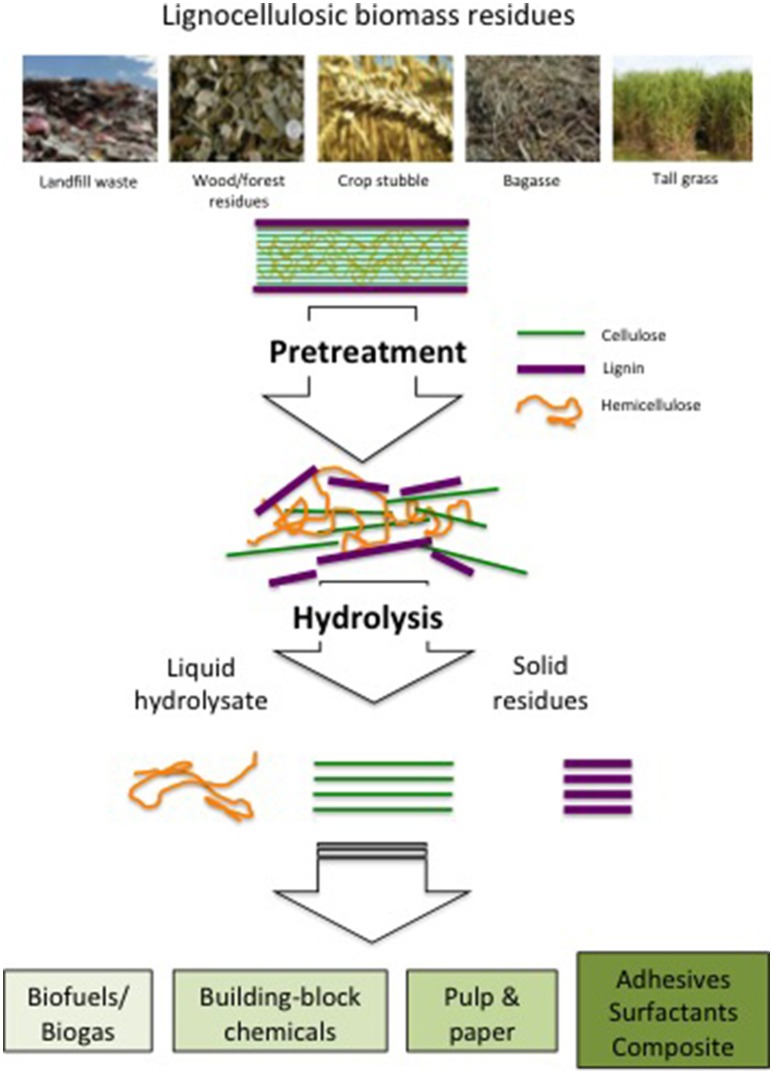
Conversion process schematics and valorization of lignocellulosic biomass residues, such as tall grass, bagasse, rice husks, wood chips, and organic landfill waste. Pretreatment of these biomass depolymerizes the complex lignin and cellulosic structures and separate lignin from cellulose and hemicellulose via hydrolysis. Subsequent fermentation and/or chemical treatment converts them into valuable energy and chemical products.

Lignocellulosic biomass generally comprises three types of biopolymers, namely cellulose, hemicellulose, and lignin. While the high polysaccharide contents in cellulose and hemicellulose make the two components valuable feedstock for bioenergy production, lignin is composed of polyphenols connected by a complex network of monomeric phenyl propanoic units with different inter-unit bonds. By cross-linking between cellulose and hemicellulose to make the cell walls rigid and three-dimensional, the complex structure of lignin constitutes the most recalcitrant component and therefore presents the greatest barrier for lignocellulosic biomass to become an economically viable energy feedstock. Industrially, lignin has been considered as a manufacturing by-product with no commercial values. For example, pulping process removes a major portion of lignin from biomass to separate the cellulosic components and generates fibrous pulp needed to manufacturing downstream products, such as papers and boards. Lignin components, on the other hand, are either combusted to produce heat value or discharged as wastewater. Consequently, many early studies on chemical and biological oxidation of lignin concerned only to removal of lignin and cellulosic residues from process effluents to comply with the environmental mandates. Lignin, the second most abundant biopolymer accounting for 15–30% of biomass, is presently limited to thermovalorization processes as filler in composites and coating materials; its chemical heterogeneity is major reason for the lack of valorization. Accordingly, pretreatment of lignocellulosic components of biomass becomes a critical step for their conversion into useful materials, as indicated in the Figure 1.

**Figure 2:**
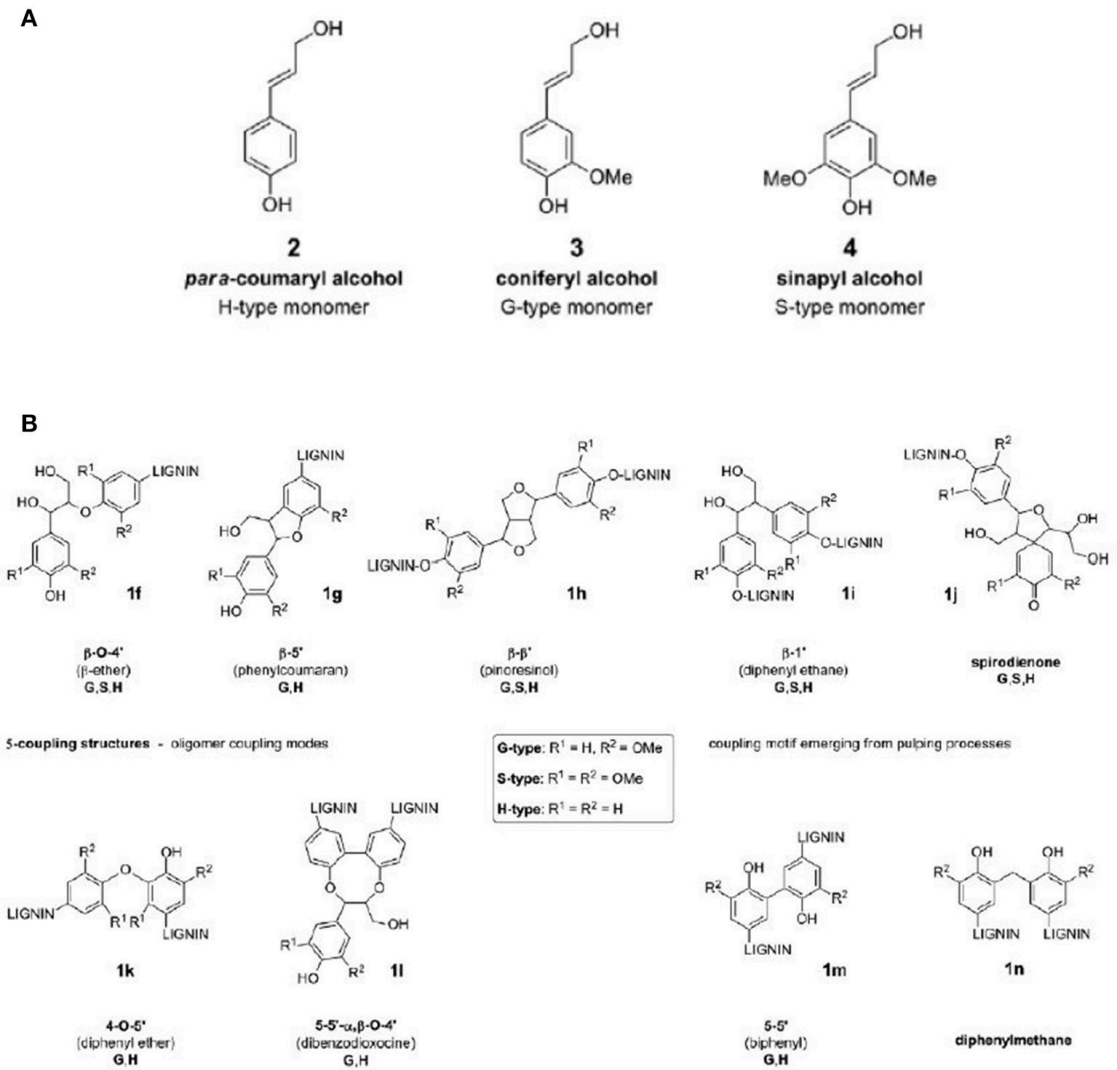
Various common bonding and linkage schemes in lignin. The figure demonstrates **(A)** three major phenylpropane units in lignin structure; and **(B)** the various types of C–O ether bonds and C–C bonds between aromatic monomer units of lignin. The figure is reproduced, without change, from Abdelaziz et al. (2016). The article is supported under the CC BY-NC-ND license (http://creativecommons.org/licenses/by-nc-nd/4.0/).

Lignin consists primarily of complex phenolic heteropolymers. The most common bonding types in lignin, as shown in Figure 2, are the β-O-4' aryl glycerol ether bond (~45%) and the β-5 phenyl coumaran bond (~10%), among others (Crestini et al., 2010). Consequently, the lack of specific bonding and subunit patterns in a lignin structure makes it challenging to decompose into selected desired compounds. Lignin is a major barrier to the use of lignocellulosic biomass in biological conversion process to bioenergy. In general, softwood possess the highest lignin matter, followed by hardwood and grasses. The intertwined spatial network of lignin is formed from three major phenylpropane units, namely syringyl (S), guaiacyl (G), and p-hydroxyphenyl (H). These units differ in the number of methoxyl groups on the benzene's aromatic backbone. Softwood lignin is primarily comprised of G (~ 90%) whereas hardwood lignin predominantly contains G and S while the grass lignin has all the three units. Since hardwood generally contains less lignin than softwood, it is technically and economically more feasible to apply anaerobic digestion process for energy conversion. Agricultural residues typically contain even less lignin (10–20% lignin, 20–30% hemicellulose, and 40–50% cellulose), and are thus more suitable as biomass feedstocks.

### Functionalities of biomass pretreatment

The intrinsic properties of lignocellulosics make it essentially non-biodegradable without any pretreatment to disrupt the lignin shield of biomass; such pretreatment helps open up the carbohydrate components so that they are accessible to microbial and/or enzymatic attacks. The changes in the physical and chemical characteristics can be detected microscopically, spectroscopically, or thermodynamically. For example, conventional scanning electron microscopy (SEM) and transmission electron microscopy (TEM) provide powerful tools to analyze the topography and distribution of lignin and cellulose in biomass samples (Fromm et al., 2003). Alternatively, environmental SEM (ESEM) enables microscopic observation of biomass samples, which are typically moist and electrically insulating, in a non-destructive manner (Turkulin et al., 2005). Furthermore, techniques using near-infrared spectroscopy have been applied to effectively quantify the individual components of lignocellulosic matrices (Xu et al., 2013; Li X. et al., 2015). Recently, taking advantage of the unique binding affinities of a luminescent oligothiophenes to lignin, cellulose and hemicellulose in various forms, a technique combining spectrofluorometric method and fluorescence confocal laser microscopic method was developed (Choong et al., 2017). This technique offers a real-time, non-destructive, visual method to analyze the chemical composition of a biomass sample during its treatment and production process.

Below is summary of pretreatment approaches used by researchers including two reviews on this topic (Alvira et al., 2010; Zhang et al., 2014).

■ Decrystallization of cellulosics: Degree of polymerization and cellulose crystallinity are important factors in determining the rate of hydrolysis. Cellulosic molecules have different orientations throughout the structure, leading to altered levels of crystallinity. In general, cellulose consists of amorphous (low crystallinity) and crystalline (high crystallinity) regions; the more crystalline the structure, the less biodegradable the molecules become. The reduction of crystallinity generally has been shown to increase biogas yield, but the inverse case may also occur. Therefore, this parameter should not be considered as the sole factor in determining a pretreatment step.
■ Reduction of lignin content: Lignin restricts the enzymatic hydrolysis rate by acting as a physical barrier thus preventing the digestible parts of the substrate to be hydrolyzed. Therefore, decreased lignin content generally leads to increased biogas yield. Treatment processes to reduce lignin contents can involve melting and lignin restructuring by steam explosion, disruption of lignin-carbohydrates linkages, or solubilization of lignin.
■ Increase in accessibility of microbes and enzymes to cellulose and hemicellulose: Accessibility of the substrate to the cellulolytic microbes or enzymes can be enhanced by increasing the available surface area for the enzymatic attack. This may be achieved by the particle size reduction of the biomass feedstock and enhancing the porosity to allow better accessibility of enzymes. Although mechanical treatment such as grinding and milling precedes other processes, nearly all physical and chemical pretreatment methods are capable of increasing the accessibility of microbes and enzymes by disrupting the hemicellulose matrix which limits the pore size in the substrate.
■ Formation of inhibiting compounds: Excessive decomposition of lignocellulosics sometimes leads to the generation of fermentation inhibitors namely furfural and hydroxymethylfurfural (HMF), either during the pretreatment step or in the ensuing anaerobic digester. For example, acidification via heated concentrated acidic solutions typically yields these inhibitory compounds that adversely affect the bioenergy conversion performance.

In summary, a successful pretreatment process ought to attain the following criteria:

- Improve the anaerobic digestibility of feedstock.
- Minimize degradation of carbohydrates to prevent conversion yield loss.
- Avoid formation of inhibitory compounds during pretreatment.
- Curtail the consumption of water, energy, and toxic chemicals.
- Reduce the process footprint (e.g., waste disposal, low toxicity, resource consumption).

### Pretreatment processes of lignocellulose biomass

The performance in terms of meeting the criteria by some of the existing pretreatment technologies is discussed hereinafter by broadly dividing the processes into those of physical-chemical methods and biological processes. It is worth noting that many of the methods involve alternation of both physical and chemical characteristics of the raw materials, hence they are generically categorized as physical-chemical processes.

#### Physical-chemical processes

##### Comminution

Comminution of biomass can be accomplished by using milling or grinding machines. Reduction of biomass particle size can modify the inherent ultrastructure of lignocellulosic biomass, increase the surface area for enzyme accessibility, reduce the degree of cellulose crystallinity, and decrease the degree of cellulose polymerization for enhanced digestibility (Kratky and Jirout, 2011). Schell and Harwood (1994) concluded that particle size must be reduced to 1–2 mm to eliminate limitations to hydrolysis; however, size reduction via mechanical forces is an expensive operation that consumes about 33% of the total electricity demand for the whole process. Another disadvantage of comminution is its inability to remove the lignin, a critical barrier to the access of microbes and enzymes to cellulose.

##### Hydrothermolysis

In this process using hot water, biomass undergoes high temperature cooking in water at an elevated pressure. During pretreatment, water can penetrate into the biomass cell structure, hydrating cellulose, solubilizing hemicellulose, and slightly removing lignin (He et al., 2016). It is an effective method for enlarging the susceptible surface area of cellulose and improving cellulose degradability, and thus has been extensively practiced to improve biogas yield from various lignocellulosic feedstock, including both energy crop (sunflower stalks, sugarcane bagasse) (Badshah et al., 2012) and biomass wastes (paper residuals, municipal solid wastes) (Qiao et al., 2011).

##### Catalytic pyrolysis

Pyrolysis refers to heating or thermally degrading organic materials in the air-free environment (Ringer et al., 2006) and may comprise a five-step process: (1) biomass heating; (2) carbonization of volatiles escaping from organic matters; (3) hot volatiles flows toward solids resulting in heat transfer to the cooler portion; (4) volatiles condensed to liquid form with incondensable gas, and (5) the autocatalytic secondary reactions (decomposition or re-polymerization). The weaker chemical bonds in lignin begin to break at lower temperatures. For examples, the hydroxyl group linked to β- or γ-carbon in aliphatic side-chain forms water as a result of fracturing. The cleavage of β- or γ-carbon bond on the alkyl side chain releases formaldehyde. Alkyl ether bond (α- or β-O-4-bond) are also cleaved. As temperature increases, stronger bonds are fractured with the exception of γ-carbon ether bond; above 500°C, the aromatic ring is depolymerized into hydrogen (Brebu and Vasile, 2010).

Conventional pyrolysis of lignin typically involves the use of catalysts, such as zeolite minerals and metallic compounds, to enhance the yields and selectivity of ensuing products from lignin pyrolysis. The metallic catalysts have been reported to increase the overall transformation of lignins into degraded products and suppress the formation of inhibitory chemicals (Maldhure and Ekhe, 2013). Other catalysts such as zeolite, are effective in promoting the cracking reactions of oxygen-containing compounds which diminish the char formation (Li et al., 2014; Kim et al., 2015).

##### Alternative activation via irradiation-microwave

Irradiation pretreatment processes pertain to alternate energy input systems via various mechanisms, such as MW, ultrasound, gamma ray and electron beam. Except for MW process, other irradiation technologies are either cost prohibitive or non-scalable; consequently, MW technology is by far the most common type of irradiation pretreatment process studied so far (Bundhoo, 2018). However, similar to traditional thermal pretreatment, MW processing can produce heat-induced inhibitors such as phenolics and furfural. Additionally, the beneficial effects of MW pretreatment of lignocellulosic biomass have not been consistently verified. For example, MW pretreatment was applied to the organic solid waste in the temperature range of 115–145°C, yielding only a 4–7% improvement in biogas production (Shahriari et al., 2012). MW treatment with temperatures of 200 or 300°C did not improve biogas production, and an increase in temperature led to lower biogas production levels (Sapci, 2013). For this reason, MW has not been used individually for lignocellulosic biomass pretreatment, but could be deployed to provide necessary heat for aiding the chemical pretreatment at relatively low temperatures without compromising pretreatment effects.

Microwave (MW) technology has been widely applied in lignin extraction from pulp industry, which substantially encourages the development of environmentally benign, innovative, and highly effective lignin conversion processes (Wang et al., 2016). Such MW-assisted technologies contain both thermal effect (i.e., fast heating) and non-thermal effect, and thus can be grouped into two major pathways: MW-assisted pyrolysis (thermal) of lignin without oxygen, and MW-assisted solvolysis (non-thermal) under milder conditions. MW-assisted pyrolysis can significantly reduce both the reaction time and temperature for lignin conversion into valuable chemicals. For example, the MW heating process for the production of bio-oil from sewage sludge has also been demonstrated using a pilot-scale MW heating apparatus (Lin et al., 2012). Maximum bio-oil yield of 30.4% was attained under the MW irradiation power of 8.8 kW and at the final pyrolysis temperature of 773°K (500°C). MW-assisted pyrolysis of various types of alkali (kraft) lignin also afforded chemical feedstock such as guaiacols (900–1240°K, 1.5–2.7 kW) and phenol (582–864°K, 700 W) (Bu et al., 2014; Farag et al., 2014).

The non-thermal effects play an important role in the chemical reactions, leading to an increase of the pre-exponential factor and a diminution of the activation energy in the Arrhenius equation. Consequently, the breaking of the β-O-4 ether bonds and C-C bonds in lignin structure can be facilitated under the MW irradiation conditions. Some studies have validated the positive effect of MW-assisted solvolysis, culminating in improved chemical yields during liquefaction processes of various agricultural residues such as wheat straw (Ouyang et al., 2015), pine sawdust (Xu et al., 2012), and bamboo (Fu et al., 2014). However, the effect of MW irradiation on the performance of biogas conversion from lignocellulosic biomass has rarely been reported.

##### Alkaline pretreatment

The method uses bases such as NaOH, KOH, Ca(OH)_2_, Na_2_CO_3_, and liquid ammonia to remove lignin, hemicellulose, and cellulose. The function of alkaline pretreatment is saponification and cleavage of lignin-carbohydrate linkages (Tarkow and Feist, 1969). Alkaline pretreatment increases porosity and internal surface area by promoting structural swelling, decreases degree of polymerization and crystallinity, thus breaking down lignin structure. NaOH is the most extensively used base to improve biogas yield from lignocellulosic biomass including agricultural residue such as wheat straw, rice straw, corn stover, woody material, and sunflower stalk. The pretreatment can be accomplished at low (0.5–4 wt % NaOH) or high (6–20 wt % NaOH) concentrations (Mirahmadi et al., 2010). Generally, at low NaOH concentration, the pretreatment aims at lignin and hemicellulose removal and is operated at higher temperature and pressure, without recycling NaOH. Contrarily, at higher NaOH concentration, process occurs at atmospheric pressure and low temperature wherein only cellulose dissolution ensues, without significant delignification. The downside of alkaline pretreatment is the possible formation of inhibitory phenolic compounds, and the higher costs of downstream processing for pH control.

##### Acid pretreatment

Acid pretreatment can be conducted either under concentrated acid (e.g., 30–70%) and at low temperature (e.g., 40°C) or under diluted acid (e.g., 0.1%) and high temperature (e.g., 230°C). Organic and inorganic acids including H_2_SO_4_, HCl, HNO_3_, H_3_PO_4_, acetic acid, and maleic acid have all been used for acid pretreatment, with H_2_SO_4_ being the most commonly used acid. The concentration of phosphoric acid beyond a critical value prompts a phase transition from swelling to cellulose dissolution (Mancini et al., 2016). The regenerated cellulose after dissolution in concentrated phosphoric acid has the dual benefit of having an amorphous form and high reactivity to cellulose (Sathitsuksanoh et al., 2012). For example, Nieves et al. (2011) achieved 40% enhancement (283 mL CH_4_/g VS) of the methane yield by employing concentrated H_2_SO_4_ (85.7 wt %) for the pretreatment of oil palm empty fruit bunches within 30 days of anaerobic digestion. Nevertheless, a pretreatment of the identical initial substrate with 8 wt % NaOH ensued in a 100% improvement (i.e., 404 mL CH_4_/g VS) of the methane generation compared to the untreated material. Similar to alkaline pretreatment, the main shortcoming of employing acid pretreatment is the deployment of a corrosive reagent, which necessitates special materials for the construction of reactor and downstream neutralization.

##### Ionic liquids (ILs)

ILs are a comparatively new class of solvents, made up of organic salts with varying melting points, high thermal stability, high polarity and the main benign attribute, barely measurable vapor pressure. Some ILs are regarded as efficient and “green” solvents for dissolution of cellulosic components. They are also attractive because large amounts of cellulose can be dissolved under mild conditions with low energy inputs, and it is feasible to recover nearly quantitatively the used IL and leave minimum residues for the downstream process (Heinze et al., 2005). The dissolution mechanism includes the oxygen and hydrogen atoms comprising cellulose hydroxyl groups, which form electron donor/electron acceptor complexes intermingling with the ILs. The cleavage of hydrogen bonds leads to an opening of the lignocellulosic network, culminating in cellulose dissolution (Feng and Chen, 2008). For example, Gao et al. (2013) showed that the lignocellulosic structure and composition were chiefly modified by the pretreatment with the IL [i.e., a mixture of 1-*N*-butyl-3-methyimidazolium chloride ([Bmim]Cl)/dimethyl sulfoxide (DMSO)], ensuing in a 28% increase of the cellulose matter in the regenerated water hyacinth and a 49% removal of lignin, with a final biogas production of 170 mL/g VS; equivalent to an enhancement by 98% compared with the untreated feedstock. Various ILs, including *N*-methylmorpholine-*N*-oxide monohydrate (NMMO), 1-n-butyl-3 methylimidazolium chloride (BMIMCl), 1-allyl-3-methylimidazolium chloride, 3-methyl-*N*-butylpyridinium chloride (MBPCl), and benzyldimethyl (tetradecyl) ammonium chloride, have been studied for pretreatment of lignocellulosic biomass to enhance enzymatic digestibility (Schell and Harwood, 1994). The main shortcomings of ILs are the associated higher costs and their inhibitory effect on the hydrolytic enzymes, even at lower concentrations. In particular, chloride bearing imidazolium cations can cause problems of corrosion and toxicity. Furthermore, the total removal of ILs after the pretreatment step requires the use of large amounts of water and involved recycling systems, which could make the process economically unsustainable (Nguyen et al., 2010).

##### Organosolv

Pretreatment of lignocellulosic materials with a mixed aqueous-organic or an organic solvent at elevated temperatures (i.e., 100–250°C) comprise the organosolv process which depends on the chemical collapse of the lignin macromolecule by breaking of ether linkages and its successive dissolution (McDonough, 1993). The most deployed solvents often used in this process are ethanol, methanol, acetone, organic acids (such as formic and acetic acid) (Papatheofanous et al., 1995) and higher boiling alcohols. Mirmohamadsadeghi et al. (2014) used organosolv pretreatment (75% ethanol at 150 and 180°C for 30 and 60 min, respectively) on three different lignocellulosic biomass varieties; 55 days of anaerobic digestion, 54.6, 79.5, and 135.2 mL CH_4_/g VS ensued from pinewood (softwood), Elmwood (hardwood) and rice straw, respectively. Methane production increased by 84, 73 and 32% with pinewood, elmwood and rice straw, respectively, in relation to the corresponding untreated entities, showing that the organosolv pretreatment is more efficient for biomass with higher initial lignin contents. Organosolv mixtures are combined with acid catalysts (H_2_SO_4_, HCl, oxalic- or salicylic acid) to cleave hemicellulose bonds in some other studies. Removal of solvents from the system is essential using suitable extraction and separation techniques (e.g., evaporation and condensation), as their recycling would reduce the operational costs; solvent separation is crucial because they might be inhibitory to enzymatic hydrolysis and fermentative microorganisms (Sun and Cheng, 2002).

##### Wet oxidation

Wet oxidation in its broad sense includes the use of oxidizing agents such as oxygen, ozone, and hydrogen peroxide. The presence of oxygen can increase the reaction rates and production of free radicals; these processes are usually performed under high temperature (125–300°C) and pressure (0.5–20 MPa). Although faster reaction rates can be achieved with high oxygen concentrations, using pure oxygen results in high operating costs. When applying a wet oxidation process to lignocellulosic biomass, all three components are affected. Hemicellulose is extensively broken into monomeric sugars and degraded into organic acids, cellulose is partly degraded, and lignin undergoes both cleavage and oxidation (Hendriks and Zeeman, 2009). Consequently, wet oxidation increases the accessibility of cellulose to biological attack via the removal of lignin and hemicellulose; pretreatment with H_2_O_2_ alone achieved 50–120% higher methane yield from rice straw (Song et al., 2012) using 1–4% H_2_O_2_, and 33% improvement for sunflower (Monlau et al., 2012) using 4% H_2_O_2_. Cesaro and Belgiorno (2013) studied the effect of ozonolysis pretreatment on biogas production from organic municipal solid waste wherein an ozone dose of 0.16 g O_3_/g TS achieved the highest biogas yield, which was 37% more than that of untreated material. Ozonolysis is a milder process as it is generally carried out at ambient temperature and pressure, and does not generate inhibitors such as furfural and soluble aromatic compounds derived from lignin oxidation unlike other wet oxidation methods. However, the non-selective nature of all wet oxidation process bears the risk of significant loss of organic matter (e.g., hemicellulose).

#### Biological processes

Fungal treatment of lignocellulosic materials for paper production is known historically. This environmentally friendly approach has received renewed attention as a pretreatment method for enhancing enzymatic saccharification of lignocellulosic biomass; cellulose is especially more recalcitrant to fungal attack than other components. Several fungi classes, including brown-, white-, and soft-rot fungi, have been used for pretreatment of lignocellulosic biomass for biogas production, with white-rot fungi (e.g., *Phanerochaete chrysosporium, Ceriporia lacerata, Cyathus stercolerus, Ceriporiopsis subvermispora, Pycnoporus cinnarbarinus*, and *Pleurotus ostreaus*) (Kumar and Wyman, 2009) being the most effective (Gao et al., 2013). For example, a wood-decaying fungus (*Auricularia auricula-judae*) was applied to pretreat sweet chestnut leaves and hay for 4 weeks; pretreated mixture of leaves and hay in 1:2 ratio resulted in a 15% improvement of biogas production. Amirta et al. (2006) employed four fungi to pretreat Japanese cedar wood chips, and found that a strain of *Ceriporiopsis subvermispora* produced the highest methane yield that was four times higher than that of the untreated sample. In general, bio-pretreatment processes offer advantages such as low-capital cost, low energy, no chemicals requirement, and mild environmental conditions. The main drawback is the low hydrolysis rate attained in most biological protocols in comparison with physical and chemical processes.

Apparently, no pretreatment processes applied to lignocellulosic biomass are optimal. The process of choice depends on the types of the biomass treated, as well as the cost effectiveness of the process. Nevertheless, an improvement of 50% pertaining to biogas yield appears to be a reasonable goal for a functional biomass pretreatment step. To attain this objective, the pretreatment will not only improve the anaerobic digestibility of feedstock, but must also minimize degradation of carbohydrates content and avoid formation of inhibitory compounds.

## Greener oxidative processes for biomass conversion-selectivity and mechanism

Greener endeavors as applied to biorefinery concept for lignocellulosic biomass conversion can be broadly classified in to two types namely, biomass transformation into energy products, and generation of valuable chemicals from biomass-derived components (e.g., lignin, cellulose, and hemicellulose). For bioenergy conversion, the key criteria for biomass pretreatment are to effectively decompose the complex lignocellulosic matrix, yet preserving the sugar content while preventing formation of inhibitory compounds to the ensuing enzymatic hydrolysis and fermentation process. In contrast, the ability of an oxidative process to selectively produce target compounds from a biomass-derived component becomes a critical criterion. For example, lignin contains a wide range of monomeric and polymeric phenolic compounds like vanillin, syringaldehyde, *p*-hydroxybenzaldehyde, vanillic acid, acetovanillone, syringic acid, among others. However, these compounds exist in low concentration amidst a complex mixture of phenolic compounds that ensue from lignin oxidation. Most of these phenolic compounds share similar molecular weights, acid dissociation constants in water, densities and melting points thus questioning their industrial application at the present time in view of the effective separation and purification challenges. Consequently, milder oxidation processes with high level of selectivity and reasonable yields are of higher research priority.

### Ozonolysis

Ozone has been accepted as an environmental-friendly oxidant with excessive oxidative power capable of non-selectively decomposing organic substrates especially for compounds containing conjugated double bonds and functional groups with high electron densities (von Gunten, 2003). Ozone is unstable in water and the pH of the water is important as the decomposition of ozone in alkaline medium results in highly reactive hydroxyl radicals (^•^OH) which is a powerful oxidizing agent with an oxidation potential of 2.33 V (vs. the standard hydrogen electrode, SHE). Therefore, ozone exhibits a faster rate of oxidation reaction for lignin degradation as compared to the conventional oxidants such as hydrogen peroxide or potassium permanganate (Kreetachat et al., 2007; Sharma and Graham, 2010). Recently, kinetics and mechanism of the oxidation of organic compounds by ozone has been discussed in detail (Lee et al., 2017).

Ozone attacks lignin more than cellulosic and hemicellulosic components of lignocellulosic material and releasing soluble compounds of lower molecular weights such as organic acids (Mulakhudair et al., 2017). Traditionally, ozonation has been used mostly for pulp “bleaching” process. The decomposition of lignin in remediating wastewater generated from pulp and paper industry and textile industry via ozonation mineralizes the complex chemicals in the effluents. However, the higher cost of ozone generation has hindered its acceptance as an oxidant in the primary treatment alternative even when the organic content of the wastewater is highly recalcitrant.

Though early studies on ozone delignification sporadically existed in the 1980's, the potential of ozonolysis for the pretreatment of lignocellulosic biomass prior to enzymatic hydrolysis and in the subsequent fermentation processes has picked up steam. The high oxidative power for decomposing the lignin component, the ease of onsite generation and utilization of ozone, and the non-inhibitory reaction products of ozonolysis, are some of the major reasons that render this greener oxidant as an attractive biomass pretreatment alternative. Ozonolysis of various biomass has been reported in the past decades, that includes cereal straws (Al jibouri et al., 2015; Garcia-Cubero et al., 2016), wood and sawdust (Mamleeva et al., 2009), sugarcane bagasse (Barrerra-Martinez et al., 2016), grass, and cotton stalks (Kaur et al., 2012; Travaini et al., 2016); the comprehensive progress made on ozonolysis of lignocellulosic biomass has been reviewed recently (Travaini et al., 2016).

For ozonolysis to be effective in structural alteration of lignin, several operating factors need to be understood and controlled especially depending on the type of biomass to be treated. These elements principally include the moisture content (MC) of the biomass, the ozone dosage, and the removal of ozonolysis by-products. Biomass MC plays a critical role in the ozonation performance, as the distance between carbohydrate fibers increases in the presence of moisture, causing biomass to swell to a different extent; swelling can expose a greater surface area with reactive functional groups subject to ozone reaction, thus enhancing the degree of lignocellulosic ozonolysis. As water content increases near or beyond fiber's water saturation, one may observe conflicting results because a typical gaseous-phase ozonation becomes more complicated as dissolution into water creates an aqueous-phase reaction with the biomass surface. For example, in the study with Aspen sawdust, the wood sample was moisturized by adding water vapor to air-dried samples to create a MC ranging between 8 and 160% w/w; at near saturation, the swollen wood contained abundance of pores filled with water molecules condensed in wood capillaries wherein the dissolved ozone allowed for a greater kinetics of ozone consumption and a faster reduction rate of lignin content. Also, as opposed to attacking the lignin component by the soluble fraction of ozone, gaseous ozone tends to react with hemicellulose components and the delignified products. Thus, an extended retention of water was favorable for more complete lignocellulosic oxidation by ozone. Conversely, in using ozone to treat ground wheat and rye straw, Garcia-Cubero et al. (2016) reported a reduction of acid insoluble lignin content (AIL) (i.e., lignin fraction resistant to enzymatic hydrolysis) as MC increased up to 30%, but further moisturization did not improve AIL reduction.

The major setback for ozonolysis of biomass pretreatment are the higher ozone generation costs, and the poor energy balance stemming from the energy required to produce ozone (2.38 MJ per 100 g O_3_) as compared to the energy ensued (2.67 MJ per 100 g ethanol). Other than continuing to develop more energy-efficient ozone generation technology, several options could make ozonolysis more competitive, including reduced ozone dosage for biomass pretreatment, improved ozonolysis efficiency for energy conversion, and identifying value-added chemicals that could be generated from ozone-pretreated biomass.

Individual studies on the effect of ozone dosages typically report ozonolysis performance using a range of ozone concentration, while other parameters are fixed. Therefore, it is difficult to compare their performance without an equal basis of ozone loading rate and other environmental conditions. Nevertheless, Barrerra-Martinez et al. (2016) in their ozonolysis study of alkaline lignin solution (1.0% w/v) and wetted sugarcane bagasse (1 g in 1 ml distilled water), found that even with a very low ozone concentration (<0.056% v/v), noticeable changes in lignin structure and enhancement in its solubilization occurred. The increase in lignin dissolution corresponded well with the results of the ensuing acid and enzymatic saccharification experiments for the bagasse sample ozonated for 90 min. In another study using low-dose ozone (<3.5% w/v) to treat steam-conditioned Norway spruce slurry, Cavka et al. (2015) reported that increases in ozone dosage led to a reduction of phenolic content and a proportional increase of acid content. The steam-generated inhibitors such as furfural and HMF, were also proportionally reduced with increased ozone dosage. In conclusion, low ozone dosages were beneficial for the fermentation of steam-pretreated Norway spruce, while high dosages decreased the inhibition of cellulolytic enzymes by soluble components in the sample. These studies demonstrated that low ozone concentration can still be effective for delignification of biomass, though the optimum ozone dosage to be applied largely depends on whether enzymatic saccharification or microbial fermentation follows.

**Figure 3:**
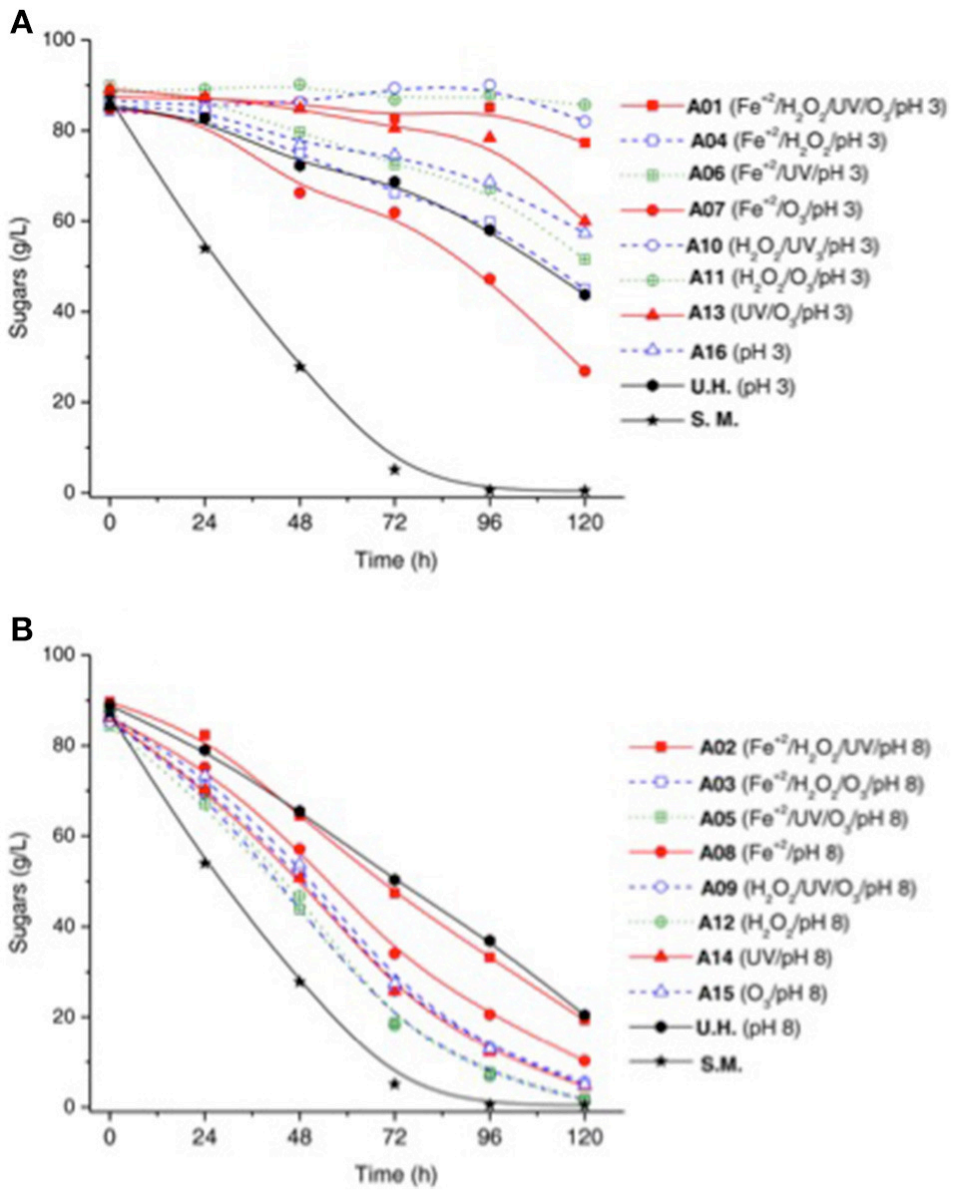
Sugar consumption by *Pichia stipites* in the hydrolysate treated in different conditions of experimental design. Sugar consumption during the fermentations by *P. stipitis* in semi-synthetic medium (S.M.), untreated rice straw hemicellulosic hydrolysate (U.H.) and the hydrolysate treated by AOPs homogeneous under different conditions of the experimental design, where: **(A)** treatments performed at pH 3, **(B)** treatments performed at pH 8. The standard deviation was <5% of the mean values of sugars consumption (Reproduced from Silva et al., 2013).

Silva et al. (2013) compared the detoxification performance of several combinations of advanced oxidative processes including H_2_O_2_, UV-C, O_3_, and Fe^2+^, under acidic (pH 3) and alkaline (pH 8) conditions, for rice straw hydrolysate conditioned with sulfuric acid and heat. The conditioned hydrolysate contained a wide spectrum of mono- and poly-phenolic compounds, including vanillin, coumaric acid, vanillic acid, syringic acid, hydroxybenzoic acid-compounds that are valuable building block chemicals but potentially inhibitory to microbial metabolism in the fermentation process to convert sugar content into bioethanol. Results are presented in Figure 3 (Silva et al., 2013) which depicts the role of varying treatment conditions on the sugar (glucose and xylose) depletion by the yeast *P. stipitis* in hydrolysates. It appears the pH was a critical factor in affecting hydrolysate fermentability. As shown in Figure 3A, the acidic pH 3.0, yeast cultured in hydrolysates treatment did not show improved sugar consumption compared to untreated hydrolysates except treatment A7 (e.g., yeast cultivated in assays A4, A6, A13, and A16 had similar or lower sugar consumption compared to those cultivated in the untreated hydrolysate. Significantly, no sugar consumption in assays A1, A10, and A11 was seen (Figure 3A). Under alkaline condition (i.e., pH 8.0), hydrolysate treatment ensued in sugar degradation (Figure 3B). Assays A3, A5, A9, A12, A14, and A15, consumption of sugar was more than 80% in 96 h.

Results from the aforementioned study suggest that hydroxyl radicals have higher affinity to aromatic rings, which contain much greater electron density than sugar molecules in the lignocellulosic matrix. Additionally, oxidation conditions in the presence of ozone resulted in the greater reduction in the total phenolic concentration (35–47 %), total furan (33–55 %), and low-molecular-weight phenolic compounds (60–96 %). In particular, ozone-based oxidation (Fe^2+^/H_2_O_2_/O_3_, and Fe^2+^/UV/O_3_) were the most effective experimental groups in alkaline pH, which generate phenolate ions due to dissociation of the hydroxyl proton of benzene rings, thus increasing their electron density and becoming more susceptible to hydroxyl free radical attack. Hydrolysate samples treated under oxidation conditions afforded the highest value of ethanol volumetric productivity of 0.36 g/l-h with a yield efficiency as high as 98% (against theoretical maximum content of fermentable sugars) using *Pichia stipitis* as the yeast inoculum for fermentation.

Alternatively, process design can improve the effectiveness of ozonolysis for energy conversion as exemplified by Al jibouri et al. (2015) in a two-step process for pretreatment of humid wheat straw; an intermediate washing procedure was sandwiched between two ozonation processes which was critical to re-exposing lignin functional groups by washing away organic acid molecules generated from the initial ozonation period, thus sustaining the effectiveness of the process. Under an optimized condition with initial MC of 45%, ozone concentration of 3% wt., washing start time of 20 min after initial ozonation and washing time of 80 s, the fermentable sugar yield by the ensuing hydrolysis process improved by 30% as compared to a one-step ozonolysis without intermediate washing, and 4 times better than untreated wheat straw. Since MC exceeding 45% adversely affected the ozonolysis performance, it is prudent to adjust the MC of the washed sample in the second-stage ozonation to 45% or less.

Adapting the multi-step pulp bleaching sequence, (Perron et al., 2016) studied the individual effect of applying ozonation, alkaline washing, and ultrasound radiation in sequence to enhance the enzymatic hydrolysis of sugarcane bagasse. The ozone feed rate was 32 mg/min for 60 min to 20 g of dry sample moistened with 10 ml of water, whereas the 0.1 M NaOH was used to for alkaline washing. They reported that ozonation alone yielded 0.8 ± 0.2 mg/g of soluble fraction in total phenolic content, whereas ozonation followed by alkaline washing significantly improved the yield up to 6.7 ± 0.1 mg/g. Correspondingly, the glucose and xylose yields after enzymatic hydrolysis for samples pretreated with ozonation alone were 16 and 12%, respectively, whereas those treated by ozonation and alkaline washing were 85 and 38%, respectively. The effect of ultrasonication as the final stage further improved the glucose and xylose yields to 94 and 55%, respectively though the extent of sugar yield enhancement was less obvious. The higher yields of fermentable sugar suggested that the initial ozonation step was a mild deliginification process while maintaining the cellulose component. Ultrasonification was not effective in improving the yield performance and was energy intensive, though its potential role as an initial pretreatment technique was not deliberated.

### Photocatalysis

Photocatalysis is considered one of the most sustainable processes for various applications because it initiates selective oxidation by harvesting abundant light energy (Ong et al., 2018; Patnaik et al., 2018). In general, photocatalysis can be provoked by photo-sensitization and photo-excitation. Photo-sensitizing molecules can be activated by absorbing ultraviolet or visible region of electromagnetic radiation, gaining sufficient energy to ionize the molecule and then transferring it to the adjacent molecules. For lignocellulosic biomass pretreatment, it has the potential to generate milder decomposing conditions and execute selective depolymerization of biomass-derived molecules into valuable chemicals. Nguyen et al. (2014) demonstrated a room-temperature lignin degradation strategy consisting of a chemoselective benzylic oxidation of a lignin model compound containing a β-O-4 linkage, followed by a photocatalytic reductive C–O bond cleavage, using an Ir-based photocatalyst to efficiently afford benzoic acid and other products. Alternatively, Gazi et al. (2015) developed a pathway to selectively initiate C-C cleavage of the same lignin model compounds using a milder and naturally more abundant vanadium oxo complex irradiated with visible light (>420 nm); pathway converted the model compound into products including an aryl aldehyde and an aryl formate, valuable building block chemicals in organic synthesis.

In photo-excitation, light energy equal or greater than the band-gap energy of a solid-state material promotes an electron (e^−^) and simultaneously creates a hole (h^+^) from the valence band. In the presence of water moisture, e– and h+ migrate along solid surface and react with water molecules to generate strong oxidizing radicals such as hydroxyl radicals (HO·), superoxide radicals (${\text{O}}_{2}^{-}\cdot$), and hydroperoxyl radicals (HO_2_·). Among an array of semiconducting metal-oxides, titanium dioxide (titania) remains the most studied material forming the basis of photocatalytic oxidation, stemming from the extended experience from titania-based application of wastewater purification containing lignin compounds (Ohnishi et al., 1989; Ksibi et al., 2003; Chang et al., 2004). Yasuda et al. (2011) studied the effect of the TiO_2_ pretreatment of two types of tropical grass on the ethanol conversion via enzymatic saccharification (Acremozyme cellulase) and fermentation (Saccharomyces cerevisiae). Photocatalytically pretreated grass samples improved the reaction rate of both the enzymatic and fermentative processes, but the conversion yield of hemicellulose into ethanol did not change noticeably.

Lu et al. (2014) depolymerized pulverized rice husks (< 180 μm) using TiO_2_ photocatalysts irradiated by ultraviolet light with a primary emitting wavelength ~365 nm in 30% peroxide solution. The depolymerized products extracted from both the aqueous solutions and solid cakes, under various photocatalytic exposure times, produced a wide array of soluble organic products including alkanes, alkenes, arenes, alkanols, alkenols, phenols, alkanals, alkenals, benzaldehydes, ketones, carboxylic acids, alkanoates, phthalates, and other nitrogen- and sulfur-containing organic compounds. They observed that alkanes, phthalates, ketones, and carboxylic acids were relatively more abundant, but there were no apparent abundance pattern of the products correlating to the time of reaction (20–100 min). The role and effect of peroxide was not discussed.

Prevention of electron-hole recombination to sustain their oxidative activities is another challenge when semiconductor-based materials are used a photocatalysts. Providing an intermediate electrode with an external anodic bias can suppress the recombination between the photogenerated charge carriers. For this purpose, Tian et al. (2010) combined the Ti/TiO_2_ nanotubes electrode and the Ti/Ta_2_O_5_-IrO_2_ electrode into a working photoelectrochemical electrode (TiO_2_/Ti/Ta_2_O_5_-IrO_2_), and tested its applicability in lignin decomposition. Individually, the reported photocatalytic degradation of lignin on the Ti/TiO_2_ electrode, irradiated with UV (365 nm) but in the absence of the anodic potential bias, resulted in the lowest reaction rate, with a first-order rate constant of 0.0025 min^−1^. In comparison, the electrochemical oxidation on the Ti/Ta_2_O_5_-IrO_2_ by applying an electrode potential of +600 mV in the absence of the electrocatalyst yielded a reaction rate that was 3-folds greater than that of the photochemical oxidation. The photochemical-electrochemical oxidation on the TiO_2_/Ti/Ta_2_O_5_-IrO_2_ electrode returned the highest rate constant (0.021 min^−1^), demonstrating the possible synergetic effect of the combined photochemical and electrochemical oxidation; vanillin and vanillic acid were the two major oxidation products.

To improve the viability and yield efficiency of semiconductor-based photocatalysis for biomass pretreatment, efforts have been made to narrow the bandgap energy so that the light energy in the visible wavelength would be sufficient to induce photocatalysis, and to sustain the reactivity by effectively separating e^−^/h^+^. An Ag-AgCl/ZnO photocatalyst was synthesized by photo-reducing Ag^+^ to Ag0 from AgCl deposited on ZnO particles (Li H. et al., 2015). The photocatalyst demonstrated complete lignin (alkali) degradation at an initial lignin concentration <50 mg/l upon irradiation with solar light for 150 min; evolution of total organic carbon (TOC) from the solution was linearly correlated with lignin degradation. The methane and biogas production yields from lignin degradation reached as high as 184 and 325 ml/g-TOC.

Recently, Gong et al. (2017) applied titania photocatalysts co-decorated with bismuth- (Bi) and platinum- (Pt) to depolymerize lignosulfonate under an simulated solar light. They reported that Bi(1%)/Pt(1%) on TiO_2_ achieved higher lignin conversion rate than Bi(1%)-TiO_2_, Pt(1%)-TiO_2_, and pure anatase TiO_2_; major reaction products being guaiacol, vanillin, vanillic acid, and 4-phenyl-1-buten-4-ol. Neither Bi nor Pt altered the crystalline structure of the anatase TiO_2_, but their presence with TiO_2_ may play an important role in controlling the e^−^/h^+^ transfer during photocatalysis. While lignin conversion rate was insensitive to the amount of Pt and Bi on anatase TiO_2_, pH and Bi:Pt ratio markedly affected the selective yield of the oxidation products. For example, the highest guaiacol yield occurred near neutral range, whereas the maximum production of vanillic acid occurred at pH 2.5. In response to the quantitative change of the Pt and Bi modifiers, the guaiacol yield peaked when an equivalent amount (1%) of Bi and Pt was added. Yields of vanillic acid maximized, however, when the amount of Bi doubled that of Pt [i.e., Bi(2%)/Pt(1%)-TiO_2_]. The addition of Pt on TiO_2_ increased the number of active sites culminating in more effective e^−^/h^+^ separation and hence the improved lignin oxidation. The presence of Bi, conversely, helped regulate the Pt active sites and favored the selective degradation of lignin into guaiacol.

Recently, Luo et al. (2015, 2017) have prepared carbazolic porous organic frameworks (POFs) possessing varying redox potential for degrading the lignin β-O-4 models under the visible-light irradiation. They fine-tuned the redox potentials of POFs in order to achieve high efficiency to oxidize benzylic β-O-4 alcohols and high yield of reductive cleavage of β-O-4 ketones; synthesized POFs showed excellent stability and recyclability. Overall, findings obtained in this study exhibited the use of visible light POF photocatalysts to generate fine chemicals from lignocellulosic biomass.

**Figure 4:**
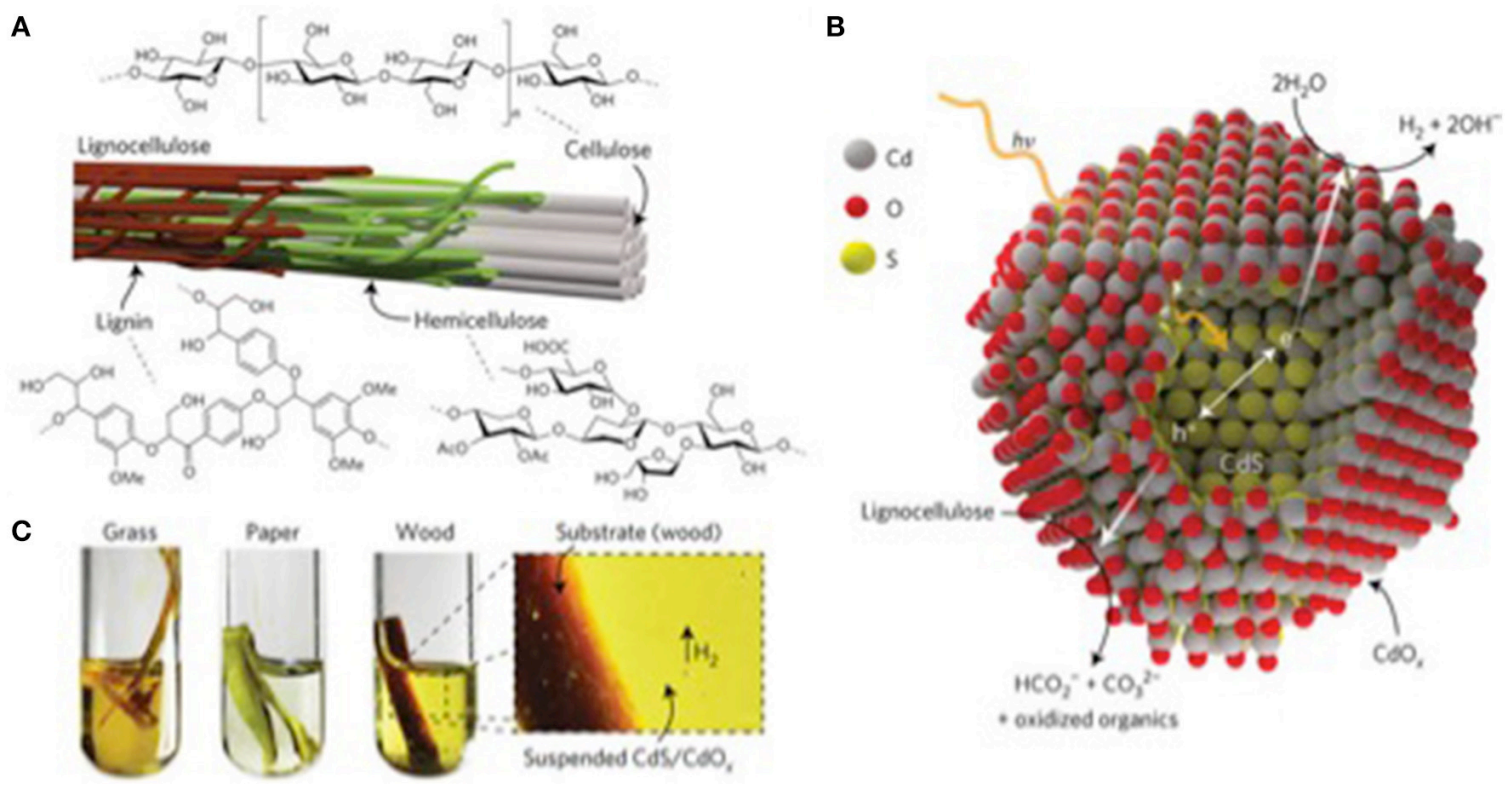
**(A)**, Lignocellulose exists as microfibrils in plant cell walls and is comprised of cellulose surrounded by the less crystalline polymers hemicellulose and lignin. **(B)**, These components can be photoreformed into H_2_ using semiconducting CdS coated with CdO_*x*_ (the CdO_*x*_ surface is believed to contain some –OH functionality, but H atoms have been removed in the illustration for clarity). Light absorption by CdS generates electrons and holes, which travel to the CdO_*x*_ surface and undertake proton reduction and lignocellulose oxidation, respectively. **(C)**, This combination creates a highly robust photocatalyst able to generate H_2_ from crude sources of lignocellulose when suspended in alkaline solution and irradiated with sunlight. The figure is reproduced with the permission of the copyright holder (Springer Nature) (Wakerley et al., 2017).

Wakerley et al. (2017) proposed a photocatalytic system based on semiconducting CdS quantum dots (QDs), which could photoreform cellulose, hemicellulose and lignin into H_2_ at room temperature (Figure 4); structures of cellulose, hemicelluloses, lignin, and lignocellulose are presented in Figure 4A). CdS is inexpensive and can absorb visible-light (a bulk electronic bandgap and potential of conduction band are ~2.4 eV and −0.5 V vs. the normal hydrogen electrode (NHE) (Yong and Schoonen, 2000). This suggests feasibility of reduction of the proton. Additionally, oxidation of saccharide is feasible due to the +1.9 V (vs. NHE) of CdS valence band (Shimura and Yoshida, 2011). This indicates that photocorrosion of CdS and evolution of H_2_ depends on applying easily oxidized sacrificial reagents (Xu et al., 2016). Wakerley et al. (2017) used highly alkaline conditions which could form Cd(OH)_2_/CdO (henceforth CdO_*x*_) on the CdS surface (see Figure 4B). Significantly, the ensuing CdS/CdO_*x*_ QDs had capacity to achieve visible light photocatalysis to generate H_2_ without photo-corrosion (see Figure 4C for the oxidation of unprocessed lignocellulosic substrates). Importantly, high dissolution of lignocellulosic at high pH could produce the synergistic effect to evolve H_2_ with high rates.

A common component from lignin, 4-hydroxy-3-methoxybenzaldehyde (vanillin), can be hydrogenated to 2-methoxy-4-methylphenol, a potential future biofuel but only at high-pressure hydrogenation of vanillin to the upgraded biofuel (2-methoxy-4-methylphenol). Formic acid, a remarkable bio-derived source of hydrogen is readily accessible from renewable resource such as sugars and their oligomers. The larger scale production of biofuels can benefit immensely from the use of formic acid as hydrogenating agent in view of its ease of transportation and as the reactions can now be conducted at ambient atmosphere. Varma and co-workers have developed a bimetallic catalyst supported on easily accessible and inexpensive graphitic carbon nitride (g-C_3_N_4_) catalyst, AgPd@g-C_3_N_4_, which accomplishes the hydrogenation of vanillin using formic acid as hydrogen source to upgrade the biofuels under visible light irradiation (Verma et al., 2016a); graphitic carbon nitride was chosen due to its ability to absorb visible light energy. The same catalyst was exploited in a novel sustainable approach to highly valuable entity, γ-valerolactone, wherein visible light mediated conversion of biomass-accessible levulinic acid occurs readily on AgPd@g-C_3_N_4_ (Verma et al., 2016b).

Photocatalysis, however, remains an inherently unselective process, especially in water and requires approaches for enhancement in selectivity that is applicable to wide ranging chemical transformations. Comprehensive and successful strategies for enhancing such selectivity in photocatalysis have been summarized recently which may help reinvigorate and stimulate future investigations (Kou et al., 2017). A recent tutorial review envisions approaches for attaining higher selectivity and yield of value-added chemicals from lignin using nanocatalysts that are embedded in the inner portions of a photomicroreactor (Colmenares et al., 2017). The development of such futuristic photocatalytic systems for lignin depolymerization in a continuous microreactor could be an exceptional approach for the production of high-value entities starting from lignin depolymerization and the fruitful accomplishment of such approaches may help in commercialization of bio-based chemicals.

### Oxidative catalysis

In recent years, several investigations have been conducted to explore different catalytic processes to obtain high value chemical from biomass. The main focus has been on overcoming the unreactive feedstock by applying acid-catalyzed depolymerization and then seeking oxidative-cleavage in the molecule. Xiong (Xiong et al., 2015, 2016) utilized bimetallic and metal carbide catalysts to have selectivity achieve C-O/C-O and C-C bond-scission; kinetic, spectroscopy, and computational approaches were applied to demonstrate the efficient biomass conversion. The synthesis of 5-hydroxymethylfurfural from carbohydrate sugars using solid catalysts has been reviewed (Agarwal et al., 2017). The formation of lactic acid from hemicellulosic biomass via a non-toxic heterogeneous catalysts-water system has been elucidated (Yang et al., 2015).

The emerging utility of polyoxometalate (POM) catalysts to convert lignocellulosic biomass to useful chemicals is noteworthy (Huber et al., 2006; Chheda et al., 2007; Alonso et al., 2010; De Gregorio et al., 2016; Romero et al., 2016; Albert, 2017; Bertleff et al., 2017; Reichert and Albert, 2017). Albert's group has shown the selective oxidation of biomass to formic acid, using tailor-made polyometalate (POMs) catalysts, termed OxFA process; the strategy entails conversion of biomass to formic acid (FA) in aqueous media under oxygen pressure without pretreatment of biomass (Albert, 2017). Studies on the conversion of well-defined substrates (e.g., glucose, cellobiose, xylose, xylose, arabinose, mannose, galactose, and cellulose) to FA using Keggin-type POMs with various degrees of metal substitution, soluble vanadium salts (NaVO_3_ and VOSO_4_), Anderson- and Wells-Dawson-type POM (Na_4_Cu_2_V_8_O_24_ and K_8_P_2_V_2_W_16_O_62_), Lindqvist-type POMs (K_2_W_6_O_19_ and K_3_VW_5_O_19_) have been performed. Keggin-type POMs had the highest yields of FA, but all model substrates could be oxidized thereby suggesting that these POMs are not suitable for fractionated biomass conversion. In case of the water-soluble vanadium precursors NaVO_3_ and VOSO_4_, same catalytic performance like Keggin-type POMs was observed thus limiting their ability for the desired conversion. The Anderson-type POM Na_4_Cu_2_V_8_O_24_ displayed no catalytic activity under the studied reaction conditions. Comparatively, the Wells-Dawson type POM K_8_P_2_V_2_W_16_O_62_ demonstrated a little undesired conversion of glucose and cellobiose. Among the various POMs, a fractionated oxidation of biomass was only observed with the Lindqvist-type catalyst K_2_W_6_O_19_, however the yield of FA was low. More research is needed to optimize the reaction conditions to achieve fractionated conversion (Albert, 2017).

The use of ionic liquids in combination with POMs has been investigated to conduct oxidative depolymerization of lignin (Cheng et al., 2014; De Gregorio et al., 2016; Ozdokur et al., 2016; Shatalov, 2016). De Gregorio et al. (2016) applied the ionic liquid (IL), 1-butylimidazolium hydrogensulfate, in combination with vanadium-based POM under oxygen rich environment (oxygen and hydrogen peroxide) to achieve oxidative depolymerization of lignin from pine and willow. Heteropoly compounds (free acids and salts of heteropolyanions, HPAs) possess high Brönsted and Lewis acidity and can perform multi-electron transfer processes efficiently. HPAs-containing homogeneous and heterogeneous reaction systems can act as bi-functional catalysts to perform oxidative delignification of lignocellulosic biomass by dioxygen (Shatalov, 2016); wood dissolution in HPAs was enhanced in ionic liquids and dioxygen facilitated the biomass delignification in this system. Role of oxygen in enhancing delignification of Southern yellow pine was also noticed in POMs-ionic liquid reaction system (Cheng et al., 2014).

Catalysts, both heterogeneous as well as homogeneous, have shown limited success in terms of cost-efficiency and productivity. The search, therefore, continues for highly acidic and relatively inexpensive and benign material which could easily convert carbohydrates to furanics in an efficient manner preferably with the possibility of total recyclability. Heterogeneous organo-catalysis appears to be an ideal choice as it is devoid of any metals. An organic sulfonated graphitic carbon nitride has been synthesized by Varma and co-workers which demonstrated its prowess in the conversion of carbohydrates to furanics and related value-added chemicals. The salient feature of the material is the high acidity and its stability which could be harnessed even at higher temperatures for cleaving carbohydrates and transforming them into biologically important scaffolds and platform chemicals (Verma et al., 2017a).

Research and development advancements in chemical processing have enabled expeditious reactions that can be accomplished in short span of time and with minimum energy requirements via innovative intensification techniques. The aforementioned photocatalytic conversion of biomass-derived materials into value-added platform chemicals (Verma et al., 2016a,b) could be improved immensely to provide a potential source of biofuels, solvents and pharmaceutical feedstocks. This could be achieved via a continuous flow reactor using the bimetallic catalyst, comprising Ag and Pd nanoparticles supported on graphitic carbon nitride surface (AgPd@g-C_3_N_4_). The sustainable feature of the approach is that formic acid used is also accessible from biomass origin and serves as the safer hydrogen source while the photoactive graphitic carbon nitride support is readily available from inexpensive urea (Tadele et al., 2017).

**Figure 5:**
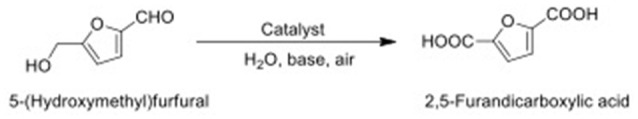
Aerial transformation of 5-(hydroxymethyl)furfural (HMF) to 2,5-furandicarboxylic acid (FDCA). HMF is typically obtained from the dehydration of lignocellulose; its conversion into FDCA provides an important polymer building block for the production of biopolymers (e.g., polyamides and polyesters).

Chitosan derived from marine waste is rich in nitrogen and is an abundant biopolymer often discarded as landfill material. A porous nitrogen-enriched carbonaceous carbon nitride catalyst (PCNx) has been prepared by Varma and co-workers using this discarded material and its utility shown in a metal-free heterogeneous selective oxidation of HMF to 2,5-furandicarboxylic acid (FDCA) (Figure 5); the process uses aerial oxygen under mild conditions to achieve highly useful conversion (Verma et al., 2017b).

### Electrochemical processes

Electrochemical process is one of the promising greener approaches for oxidative pretreatment of lignocellulosic biomass, as it is a reagent-free process (Frontana-Uribe et al., 2010) and entails electrochemical generation of oxidizing agent such as hydroxyl radical to decompose organics or polymers to their mineralization (Sires et al., 2014). It is highly dependent on type of solvent and supporting electrolyte, nature of electrode and the electrode potential (Chum et al., 1985). The process provides means of valorization of lignin to high value compounds such as fine chemicals or biofuels, an essential feature of future biorefinery industries (Tuck et al., 2012; Luque and Triantafyllidis, 2016). For instance, kraft black liquor (wastewater from wood chip washing) is particularly satisfactory for electrochemical process in view of its sufficiently high conductivity (Di Marino et al., 2016).

Electrochemical oxidation has been explored for the degradation of lignin, but it suffered from electrode fouling problem caused by polymerization. Recent efforts for the oxidation have moved forward to the development of high activity electrocatalysts (Tolba et al., 2010); nickel-based anode for electrochemical degradation of lignin resulted in yield of high-value fine chemicals like vanillin and acetovanillone (Schmitt et al., 2015). The Ni electrode exhibited high stability and little corrosion issue. 3D materials such as stainless steel net and Ni form have been employed to improve the yield of vanillin. Possible lignin degradation mechanism was proposed using titanium-based electrodes (Ti/Sb–SnO_2_ and Ti/PbO_2_) (Shao et al., 2014) wherein decomposition pathway include opening loop of quinoid structure, damage of alkyl-aryl ether bond and demethylation. This study also showed selective and non-selective oxidation models from the studied electrodes.

IrO_2_-based electrodes (Ti/Ta_2_O_5_-IrO_2_, Ti/SnO_2_-IrO_2_, Ti/RuO_2_-IrO_2_, and Ti/TiO_2_-IrO_2_) have been fabricated for electrochemical oxidation of lignin to primary products, vanillin and vanillic acid (Di Marino et al., 2016). Among all of the tested electrodes, Ti/Ta_2_O_5_-IrO_2_ showed the highest electrochemical active surface area, while Ti/RuO_2_-IrO_2_ exhibited the highest stability, long lifetime (40 days) and activity for lignin degradation. The estimated apparent activation energy for the electrode at 20 kJ/mole is similar to the energy for hydroxyl radical reactions (Labat and Gonçalves, 2008). Influence of current density on the oxidation of lignin has been investigated; increase in the current density (200–500 mA/cm^2^) showed increase in apparent rate constant, but further increase in current density resulted in oxygen evolution reaction and electricity waste. TiO_2_ nanotube/PbO_2_ electrode was used for the treatment of kraft lignin, taking the advantages of high oxidativity and increased usable surface area (Pan et al., 2012); deposition of PbO_2_ nanoparticles onto the TiO_2_ nanotube apparently enhanced the electrocatalytic activity of the electrode. The oxidation process followed the pseudo first order kinetic with estimated activation energy of about 16.04 kJ/mole; vanillin and vanillic acid being characterized as the two primary products of the process. These studies suggested that the optimization of current efficiency would greatly benefit the energy- and cost- effectiveness of the process.

Another key electrochemical technology for depolymerization or oxidation of lignocellulosic biomass is electrodialysis that utilizes electric potential as driving force to separate ions or species through an ion exchange membrane. The technique has been widely used in desalination and wastewater treatment processes, and is now a newer area of focus for bio-separation process (Lee et al., 2013). Electrodialysis using bipolar membrane has been utilized to assist acidification pretreatment to extract lignin by precipitation from the kraft black liquor (Haddad et al., 2017) which seemingly used fewer chemicals than conventional acidification pretreatment and could be used to simultaneously produce caustic soda. Electrodialysis has been investigated to integrate with oxalic acid pretreatment of waste mushroom medium (Lee et al., 2013); the method was aimed to remove inhibitory compounds from the process, which consequently increased ethanol production by about three times.

**Figure 6:**
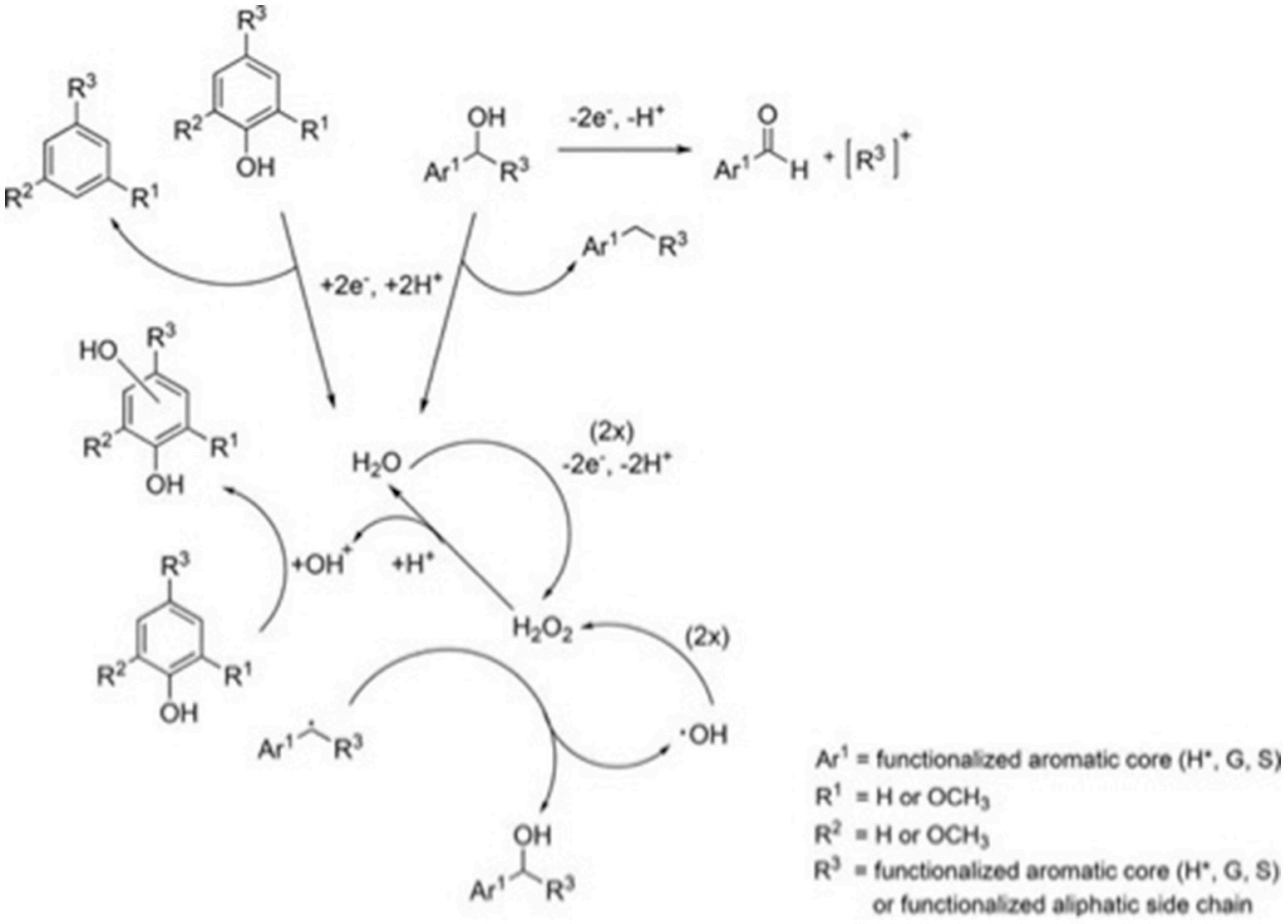
Abbreviated reaction scheme showing proposed electrochemical/radical mechanisms during lignin degradation. The numbers in parentheses give the equivalents of raw material needed for the reaction. Aromatic core units are defined as follows: (H^*^) 4-hydroxybenzyl, (**G**) 3-methoxy-4-hydroxybenzyl, (**S**) 3,5-dimethoxy-4-hydroxybenzyl. The figure is reproduced, without change, from Dier et al. (2017). The article is supported under the CC BY-NC-ND license (http://creativecommons.org/licenses/by/4.0/).

Novel approaches integrating electrochemical process with other pretreatment protocols have garnered attention for biomass processing. Electrochemical depolymerization of lignin has been achieved using the ionic liquids, (1-ethyl-3-methylimidazolium trifluoromethanesulfonate and triethylammonium methanesulfonate) (Dier et al., 2017) which could simultaneously degrade lignin and help assist in the recovery of the electrolyte material. The formation of H_2_O_2_ played an important role for the lignin degradation. A proposed mechanism of the degradation of lignin is presented in Figure 6. The formation of low molecular weight fractions proposed in the mechanism was confirmed by liquid chromatography-high resolution mass spectrometry (PC-HRMS) and gas chromatography-mass spectrometry (GC-MS) techniques.

Pure deep eutectic solvents (choline chloride with urea or ethylene glycol) coupled with an electrochemical process was investigated to dissolve and process electrochemical oxidative depolymerization of kraft lignin (Di Marino et al., 2016) wherein guaiacol and vanillin were detected as the most abundant products. Electrochemical technology has been deployed to enhance biogas formation via aerobic digestion (Song et al., 2010; Katsoni et al., 2014). Electrochemical process for pretreatment of waste activated sludge showed relatively low specific energy input with improved methane production when compared to other pretreatment methods involving microwave, ultrasound, thermal, Fenton, and ozone (Ye et al., 2016).

### Fenton and fenton-like reactions

Fenton reaction involves hydrogen peroxide (H_2_O_2_) and ferrous iron that generate highly reactive species ^•^OH [Fe(II) + H_2_O_2_ → Fe(II) + ^•^OH + OH^−^] (Ganzenko et al., 2017; Jain et al., 2017). Fenton reaction is commonly used to oxidize contaminants such as dyes, trichloroethylene (TCE) and perchloroethylene (PCE) (Diagne et al., 2014; Lin et al., 2017) and generally works efficiently in the pH range from 2.5 to 3.5. Other limitation of the Fenton reaction is the production of iron-containing sludge as secondary pollutants. To overcome this disadvantage of the Fenton reaction, heterogeneous iron-containing catalysts are applied to produce Fenton-like reactions (Barhoumi et al., 2016; Ouiriemmi et al., 2017; Steter et al., 2018); such reactions have been extensively sought to degrade and mineralize numerous organic pollutants (Feng et al., 2017; Mirzaei et al., 2017; Sharma and Feng, 2017; Li et al., 2018).

Zhang and Zhu (2016) studied the synergistic effects of pretreatment of sugarcane bagasse (SCB) using Fenton reaction and NaOH extraction on sugarcane bagasse. Sugarcane bagasse (5 g) was added to a solution of Fe^2+^ (50 mL) to make for a solid–liquid ratio of 5%. Enzymatic hydrolysis and fermentation was done using SHY07-1 yeast (inoculum dosage of 10% v/v) and glycerol stock in YPX medium. Sugar analysis along with SEM and XRD analysis was performed. The optimum Fe^2+^ concentration was found to be 20 mM and the concentration of hydrogen peroxide was fixed at 10% (w/w). Similarly, the pH and temperature were optimized to 2.5 and 55°C respectively. It was found that initial NaOH extraction followed by Fenton reaction had little or no effect on the sugarcane bagasse structure. On the other hand, Fenton reaction first followed by NaOH extraction resulted in the enhanced erosion of the structure by subsequent NaOH extraction step (de Almeida et al., 2013).

Simultaneous saccharification fermentation (SSF) is considered a process to decrease cellulase inhibition by glucose and simplify the operation via the integration of saccharification and fermentation steps, thereby increasing the fermentation efficiency (Hasunuma and Kondo, 2012). It was discovered that the NaOH extraction as compared to Fenton reaction resulted in greater ethanol production. Apparently, Fenton reaction causes high accumulation of Fe^3+^ ions which inhibit the activity of b-glucosidase culminating in less conversion of glucose to ethanol.

An ideal pretreatment step is one in which the cellulose is left unharmed while a majority of the lignin and hemicellulose are removed (Gabhane et al., 2015). In the case of Fenton pretreatment, there was an 18.3% removal of lignin component, a 16% increase in cellulose and a slight decrease in hemicellulose; total cellulose being 55.8% which was still higher than that of the raw sugarcane bagasse (52.8%). Conventional alkaline pretreatment had a lignin removal rate of 42%. NaOH extraction achieved more total cellulose content and less residual lignin in the pretreated sugarcane bagasse than Fenton pretreatment. The combination of the two methods (NaOH extraction followed by Fenton pretreatment), however, showed a synergistic performance, with about 50% of lignin removal and almost 100% increase in cellulose content. There was a large hemicellulose loss with only 6.8% remaining after the synergistic performance. When the sequence order was reversed, there was a steady increase of 59.8% in lignin removal, a slightly lower cellulose content but less hemicellulose loss.

Jung et al. (2015) studied the effect of Fenton pretreatment of rice straw on the increase in the enzymatic digestibility for the saccharification of lignocellulosic biomass wherein the Fenton's reagent (FeCl_3_ and H_2_O_2_) was used. This was the first reported application of the Fenton reaction for lignocellulose pretreatment at a modest temperature of 25°C and with a relatively higher biomass loading of 10% (w/v). The concentrations of FeCl_3_ and H_2_O_2_ were altered between 0.01–0.1 and 0.5–4.0 M, respectively, to afford varying ratios of Fe^3+^ to H_2_O_2_ in the range of 1:10–1:100. A temperature of 25°C was maintained and the mixture was shaken at 200 rpm. The various byproducts namely HMF, furfural, acetic acid, levulinic acid, formic acid and glycerol, formed in the dissolved fraction of the pretreated rice straw slurry were quantified by HPLC measurements.

For a fixed FeCl_3_ concentration, the enzymatic digestibility of rice straw pretreated with the Fenton's reagent usually enhanced with an increased H_2_O_2_ concentration possibly due to the greater oxidizing ability of H_2_O_2_ under acidic environments. Higher digestibility values, 88.7 and 87.5% of the theoretical maximum glucose yield, were obtained after pretreatment using the two diverse concentrations of the Fenton's reagents comprising 0.03 M FeCl_3_ and 2.25 M H_2_O_2_ and 0.05 M FeCl_3_ and 2.5 M H_2_O_2_, respectively. An analysis of the rice straw after pretreatment with the Fenton's reagent for 24 h showed that the amounts of both, the lignin and xylan were substantially reduced to 9.3 and 3.6%, respectively. When either H_2_O_2_ or FeCl_3_ was used as the only reagent for pretreatment, the amount of lignin was reduced to 16.2 and 15.7%, respectively. Additionally, when ~40% of the initial amounts of lignin and glucan were solubilized after pretreatment using various compositions of the Fenton's reagent, higher values of enzymatic digestibilities i.e., > 70% were observed. This significant solubilization, in conjunction with breakdown of sugar in the rice straw could be due to the indiscriminate oxidation of lignocellulose by engendered reactive oxygen species in the Fenton reaction.

The whole mechanism of the Fenton reaction in the pretreatment of rice straw is as follows. The presence of Fe^3+^ and H_2_O_2_ yield an array of reactive oxygen species, e.g., hydroxyl-, perhydroxyl radicals, organic peroxyl radicals, and iron ion conjugates namely ferrous- and ferryl-binding compounds which attack lignin and hemicellulose on the outer surface of lignocellulose, and generate demethylated, oxidized, or fragmented lignin and polysaccharides (Koenigs, 1974; Kirk et al., 1985).

Kato et al. (2014) studied the effect of Fenton pretreatment on the total organic carbon (TOC) and lignin composition of four biomass feedstocks miscanthus (*Miscanthus giganteus*), switchgrass (*Pancium virgatum*), wheat straw (*Triticum aestivum*) and corn stover (*Zea mays*). Enzymatic saccharification showed a significant increase in glucose production upon Fenton pretreatment across all four feedstocks as well as an increase in cellulose bioavailability to *in vitro* cellulase exposure. The latter suggests a reduction in the recalcitrant nature inherent to lignocellulosic materials. A lignin assay analysis was performed on all four samples which showed that there was a decrease in the acid-insoluble lignin (AISL) content in miscanthus (6.63%), corn stover (14.1%) and wheat straw (8.76%) while switchgrass (3.59%) produced no statistically significant decrease (*P* < 0.05). In the case of acid soluble lignin (ASL) content, switchgrass (22.6%) and wheat straw (16.8%) showed a statistically significant (*p* < 0.05) decrease while both miscanthus (4.61%) and corn stover (0%) showed no decrease relative to the untreated biomass. The aforementioned results show that solution phase Fenton pretreatment may not be degrading lignin as seen in *in vivo* Fenton chemistry of white-rot fungi. It is however, altering the biomass in a way that allows cellulose to be bioavailable as observed by the significant increase in enzymatic saccharification. It has been postulated that Fenton pretreatment may possibly clip or alter the macrostructure of lignin analogous to ammonia fiber explosion (AFEX), thus enabling cellulase enzymes better access to cellulose (Kumar et al., 2009). Aforementioned studies suggest that Fenton reaction can be effective in fragmenting the surface structure but less likely to selectively oxidize the lignocellulosic components into desirable chemical derivatives without other means of oxidative intervention.

## Outlook and perspective on the conversion of biomass into value-added chemicals

### Techno-economic assessment

The concept of biorefineries using lignocellulosic biomass residues as raw feedstocks has been defined and discussed extensively over the past decade in an effort to accelerate the commercialization process to provide a sustainable alternative to the growing demand of energy. Earlier studies have focused on supply chain modeling and optimization for biomass harvesting, collection, inventory, preprocessing, production, and distribution of the biofuel system to provide tools for strategic analysis and tactical planning that aimed to minimize cost of production (Zamboni et al., 2009; Kim et al., 2011; Rincón et al., 2015). This is mostly because biomass is characterized by seasonal and geographical fluctuations, hence the conventional, vertically integrated feedstock supply system where feedstock is obtained from local growers, and delivered in low-density format to the centralized conversion facility can be cost prohibitive. Additionally, feedstock supply scarcities and price instabilities due to reduced harvests and competition from other industries can also pose risks to investment and plant operation (Lamers et al., 2015). Studies have shown that the cost of pretreatment of heterogeneous biomass residues and the charges for collection, inventory and transportation can take up as much as 50% of the production cost distribution of lignocellulosic biofuels and biochemicals. Kurian et al. (2013) reviewed these aspects addressing the feedstocks supply chain and logistics system for lignocellulosic biorefineries, aiming to reduce the cost and improve profit distribution for the economical production from low density lignocellulosic biomass. One of the models to help the causes is to form decentralized depots of on-site biomass collection and preprocessing. The approach is built on the concept of having a network of biomass supply chain for the operation of a biorefinery by pretreating biomass into various intermediate products. These transitional products can meet the biomass supply for the production of biofuels and biochemicals, and the local demands for electricity and animal feed. The decentralized biomass pretreatment that densifies the feedstocks can then be transported to a centralized biorefinery for the conversion into fuels and chemicals. This strategy can effectively reduce the transportation cost and increase the production capacity, while addressing the local resource demands and environmental issues. In brief, the options to reduce negative impacts on resource and environment include: i) native sourcing of lignocellulose; ii) improving crop straits; and iii) developing a biorefinery model. Both options (ii) and (iii) can potentially aim to attain higher yield of energy and value-added products, and to reduce the resource and environmental impacts. In this regard, replacing conventional pretreatment processes involving abrasive chemicals and energy-intensive conditions with milder physical and chemical processes are in line with the future visions of the biorefinery technologies.

### Life-cycle perspective

Life cycle assessment (LCA) of environmental impacts is of particular interest for biomass-related studies. It is a fundamental analytical approach to understand the actual contributions to climate change or other environmental issues thus identifying opportunities for environmental improvements (Gnansounou et al., 2009; Singh et al., 2010). It is also an important step for sustainability improvement of green chemistry technologies before scale up or implementation (Tufvesson et al., 2013). LCA of lignocellulosic biofuel have been primarily studied for the converting biomass into ethanol. Lignocellulosic ethanol was generally reported to have lower greenhouse gas (GHG) emissions than gasoline and conventional grain-based ethanol (Gerbrandt et al., 2016). Morales et al. (2015) and Borrion et al. (2012) also concluded a clear reduction in GHG emissions and ozone layer depletion for lignocellulosic ethanol studies, with highest reduction for corn stover and wheat straw, while other impacts such as acidification, eutrophication, human health and photochemical smog were positively or negatively affected. Most of the LCA studies on lignocellulosic ethanol production appear to be energetically sustainable, with energy balance ratio (heat content of the fuel to non-renewable primary energy input for the fuel) > 1, partly due to the possibility of using by-products as fuel in a cogeneration system (Gerbrandt et al., 2016).

As most of the lignocellulosic biomass pretreatment methods are still under development, there is a limited understanding on their environmental impacts (Prasad et al., 2016). Pretreatment of wheat straw using steam explosion without acid catalyst (SE), liquid hot water (LHW) and wet oxidation (WO) were environmentally favorable over dilute acid (DA) and steam explosion with acid catalyst (SEAC), from LCA results. Pretreatments of SEAC and DA that employed sulfuric acid and ammonia as reagents resulted in comparable contributions in global warming, acidification, eutrophication, ozone layer depletion and ecotoxicity impacts (Wang et al., 2013). Similarly, for a LCA study of corn stover-based ethanol pretreated by LHW, DA, SEAC and organosolv, LHW demonstrated the highest sugar conversion rates and significant reduction in CO_2_ emissions but slightly higher water depletion rates, whereas DA was the least preferable choice due to long processing duration (12 h) and intensive electricity use (Gnansounou et al., 2009). Relatively higher acidification potential was also discerned in that study, as a consequence of significant SO_2_ emissions from electricity production for the lime pretreatment step (Gnansounou et al., 2009). Electrochemical oxidation of lignin consumes considerably low electricity and is regarded as an environmentally friendly pretreatment method (Shao et al., 2014).

The use of acidic and basic reagents as well as energy demands for processing seemed to determine the contribution of environmental impacts of conversion of lignocellulosic biomass into products. To this end, development of green and sustainable pretreatment and treatment of the lignocellulosic biomass is generally encouraged. Future efforts in energy-intensive approaches such as SEAC and DA can be improved by substituting their energy being supplied using greener or renewable energy sources (Gnansounou et al., 2009). A modified DA pretreatment for woodchip-based bioethanol was proposed to reduce environmental impacts of land use by 18.5% and ecosystem quality by 17% (Shadbahr et al., 2015). Electrodialysis was investigated for a multistage of oxalic acid-based pretreatment to recover and reuse of the acid (Pan et al., 2012). Deep eutectic solvent as an inexpensive environmentally reagent (Khandelwal et al., 2016) was investigated for selective generation of vanillin by electrochemical process (Tuck et al., 2012). Synergistic benefits have been demonstrated for combinative pretreatment systems (Kavitha et al., 2015). These propositions incorporate the green chemistry principles with improvements to prevent waste and use of hazardous materials, improve material and energy efficiencies, use of renewable resources and, design for reusing/recycling (Tabone et al., 2010).

### Socio-economic impact of valorizing biomass residue

While LCA framework and methodology provides quantitative indicators that can adequately address the techno-economic and environmental impacts of a developing technology such as lignocellulosic biofuels, the socio-economic and cultural impacts are engaging more subjective arguments given the value conflicts of any given society. For example, Malik et al. (2016) developed an input-output (IO) analysis combining LCA criteria to assess the “triple bottom line” (i.e., social, economic and environmental impacts) of lignocellulosic biofuel production in South Australia. They concluded that gains in economic activities and employment outweigh the losses, in that the biofuel industry would increase the productivity and economic growth in rural part of the regional Australia. The study also showed that the lignocellulosic biofuel industry would result in net carbon-negative (i.e., the amount of CO_2_ emitted will be less than the amount sequestered for obtaining the wood biomass), and that the energy return on investment (EROI) for all the cellulose-refining scenarios gave a net gain in energy. Diamantopoulou et al. (2016) evaluated the socio-environmental impacts of fermented biofuels with two different feedstocks (wheat bran and barley straw) in Greece by formulating a metric tool named “biomass sustainability index” (BSI) consisting of three vectors (i.e., preserving natural resource, maintaining natural cycle and ecosystem, social acceptance) that are further expanded into 12 indicators. The scoring system based on three-level (positive, negative, neutral) feedbacks from participants gives a mapping analysis how the public perceive the biofuel industry. The BSI of each of the first-generation ethanol received low scoring and did not appear sustaining as compared with ethanol generated from various biomass residues. Briefly, the study concluded that the “best-practice” scenario with barley straw as the biofuel feedstock received markedly higher score than it did with wheat bran, whereas both feedstocks received low and similar scores under the scenario of “maximum profit”, primarily attributed to perceived concern of irreversible resource and environmental harms. Neither of the feedstock BSIs showed any significantly impact on local employment and regional development, implying the stakeholders in the region did not expect a major economic contribution from the biomass-to-bioenergy industry.

To close the gap in the dimension of social impact of biofuels, Ribeiro and Quintanilla (2015) applied the Delphi survey technique to explore the perception of biofuel experts from diverse countries on prospective social impacts of cellulosic ethanol. The study devised two technological pathways to address the technical aspects of a transition of cellulosic ethanol from conventional feedstock (e.g., starchy and sweet crops) to second-generation lignocellulosic feedstock (e.g., dedicated energy crops, residues, municipal organic solid waste). The participants were surveyed for their opinions on the potential impacts pertaining to social dimension (e.g., negative impacts on food security, the inclusion of small-scale farmers, exclusion of low-skilled workers, exclusion of small-scale producers in the supply chain) and environmental dimension (e.g., negative impacts on water security, negative impacts on biodiversity security) with several scenarios involving different regions, type and source of feedstocks. The opinions of expert panelists indicated that using non-edible raw material in place of food crops to produce cellulosic ethanol might not be the answer to overcome food security concerns. They considered the lignocellulosic feedstock for ethanol production rests in the same agricultural paradigm to that of conventional ethanol. However, the use of municipal solid waste and residues as feedstock for cellulosic ethanol production was considered favorable over the use of selected energy crops. Also, given that cellulosic ethanol is still being produced on an experimental stage, the contribution of cellulosic ethanol to rural development (supply chain comprising predominately small landholders and small-scale producers) is uncertain. This study shows that experts were skeptical on the prospect of transitioning bioethanol production from convention to lignocellulosic feedstocks, especially when production is based in poorer countries.

To bring the debate of social value into more systematic discussion, Raman et al. (2015) applied the framework of responsible innovation, which emphasizes developing a shared understanding of how to bring forth societal and value questions on a technology innovation, to sustainability assessment. The value-based questions, such as the valuation of land, of biomass, and of nature, often dictate how the members of a regional-specific society assess the potential impacts of lignocellulosic biofuels on environment (i.e., GHG emissions, biodiversity, water, soil, and air) and access to resources (i.e., food, water, energy). These potential impacts by the life-cycle components (e.g., crops, residues, processing, conversion, and distribution) can be individually identified and evaluated in either global scope or regional scope. Furthermore, the basis with which the core values are compared must be defined. For example, the core value pertaining to the techno-economic proficiency of lignocellulosic biofuels is compared with the first-generation biofuels and fossil fuels, whereas the yardstick for socio-economic justice is the impacts on global agricultural economy as compared to the existing system. One such argument is that, while first-generation biofuels imposes concerns on food competition, the question of whether producing second-generation biofuels (lignocellulosic biofuels) would distribute socio-economic impacts more fairly than the first-generation biofuels (or fossils) lingers. This is because biomass is characterized by seasonal and geographical variations, reflecting that lignocellulosic biomass residues could be an indispensable source of energy, food, animal feeds, or other uses in different regions of the world, especially in the developing countries. In summary, each stakeholder inherently prioritizes its own interests and needs, adding a great deal of uncertainties to the steady supply of biomass residues (Perez et al., 2017). Therefore, even with multitude of drivers, commercializing biofuel production from lignocellulosic biomass does not guarantee the eventual economic benefits for farmers or biomass producers, unless a well-thought management and logistics are implemented.

## Author contributions

WD took the lead in coordinating the review study and drafting the manuscript. WD, VS, RV, ML, and GN all performed critical reviews and contributed to the writing of sections Greener Oxidative Processes for Biomass Conversion-Selectivity and Mechanism. ML also contributed part of section Outlook and Perspective on the Conversion of Biomass into Value-Added Chemicals. RV and VS critically revised the article and helped shape the final version of the article. All authors provided comments on the manuscript and approve the final version to be published.

### Conflict of interest statement

The authors declare that the research was conducted in the absence of any commercial or financial relationships that could be construed as a potential conflict of interest.

The reviewer, AB, and handling Editor declared their shared affiliation.
